# Factors Associated with Progression to Preeclampsia with Severe Features in Pregnancies Complicated by Mild Hypertensive Disorders

**DOI:** 10.3390/jcm12227022

**Published:** 2023-11-10

**Authors:** Sivan Barda, Yochai Yoeli, Nitzan Stav, Amir Naeh, Esther Maor-Sagie, Mordechai Hallak, Rinat Gabbay-Benziv

**Affiliations:** Department of Obstetrics and Gynecology, Hillel Yaffe Medical Center, The Rappaport Faculty of Medicine, Technion—Israel Institute of Technology, Haifa 3200003, Israelamir_naeh@outlook.com (A.N.); estigal02@yahoo.com (E.M.-S.); mottih@hymc.gov.il (M.H.)

**Keywords:** preeclampsia, severe features, gestational hypertension, superimposed preeclampsia, mild features, progression, pregnancy complications, prediction

## Abstract

In this retrospective cohort study, we aimed to investigate the variables associated with progression to preeclampsia with severe features in parturients already diagnosed with mild hypertensive disorders of pregnancy. The study was conducted in a single university-affiliated medical center between 2018 and 2020. All women admitted due to hypertensive disorders were included. Data collected was compared between parturients who progressed and did not progress to preeclampsia with severe features. Among 359 women presenting without severe features, 18 (5%) developed severe features, delivered smaller babies at lower gestational age, and with higher rates of cesarean delivery (*p* < 0.001 for all). Chronic hypertension, maternal diabetes, any previous gestational hypertensive disorder, gestational diabetes, number of hospitalizations, earlier gestational age at initial presentation, and superimposed preeclampsia as the preliminary diagnosis were all associated with preeclampsia progression to severe features. Previous delivery within 2–5 years was a protective variable from preeclampsia progression. Following regression analysis and adjustment to confounders, only gestational age at initial presentation and superimposed preeclampsia remained significant variables associated with progression to severe features (aOR 0.74 (0.55–0.96) and 34.44 (1.07–1111.85), aOR (95% CI), respectively, *p* < 0.05 for both) with combined ROC-AUC prediction performance of 0.89, 95% CI 0.83–0.95, *p* < 0.001. In conclusion, according to our study results, early gestational age at presentation and superimposed preeclampsia as the preliminary diagnosis are the only independent factors that are associated with progression to severe features in women already diagnosed with mild hypertensive disorders during pregnancy.

## 1. Introduction

Hypertensive disorders of pregnancy are one of the leading causes of maternal, fetal, and neonatal morbidity and mortality worldwide [1], with an overall incidence ranging from 4 to 25 % [2], and an increment in incidence in the last few decades [3,4]. Overall, 10 to 15 percent of direct maternal deaths are attributed to preeclampsia (PE) [5], and in the United States, PE is one of the four leading causes of maternal death [6]. The pathophysiology of PE is multifactorial and includes trophoblast immaturity [7], increased oxidative stress [8], and vascular dysfunction [9].

Maternal-wise, morbidity and mortality are mainly related to severe hypertension, systemic inflammation, and end-organ failure (e.g., renal failure, cerebral hemorrhage or stroke, pulmonary edema, impaired liver function, retinal injury, and hematological dysfunction) that accompany PE with severe features (sPE). Fetal and neonatal wise, most complications are related to early PE and preterm delivery associated with growth restriction and prematurity [10].

Nevertheless, some complications such as abruption and fetal demise can occur even with term PE. Moreover, PE is a progressive disorder, and women with any type of hypertensive disorder during pregnancy can progress to sPE and be subjected to any of the above complications. Be that as it may, and despite growing incidence, most PE cases occur at late gestation with a generally favorable maternal and neonatal outcome and complete resolution of the disease within a few days after delivery.

The variable course of PE and other mild hypertensive disorders of pregnancies, and the association with both short-term and long-term morbidity and mortality if the disease progresses to severe features [1,2,3], raises the importance of recognizing the parturients that will eventually progress to sPE and may benefit from close monitoring of blood pressure, repeated labs, fetal assessment, and antenatal steroids.

Despite numerous studies that evaluated the risk factors and constructed models to assess the risk for PE, especially prior to or in the first trimester of pregnancy, only a few publications evaluated the risk for sPE among parturients already diagnosed with hypertensive disorders of pregnancy [11,12,13,14]. Therefore, in this study, we aimed to evaluate the risk factors that are associated with progression to severe disease in parturients already diagnosed with mild hypertensive disease during pregnancy.

## 2. Materials and Methods

This was a retrospective cohort study that included all women who delivered in one university-affiliated medical center, between 1 January 2018, and 31 December 2020. We searched our computerized comprehensive perinatal database for all women who were admitted after 20 weeks of gestation with any hypertensive disease of pregnancy—gestational hypertension (GH), PE, or superimposed PE according to ICD-9 diagnosis (642.3x–642.7x). Women were excluded if delivered elsewhere, if the delivery date was missing, or if, at first presentation, were defined as sPE. The study was approved by the local Institutional Review Board committee (HYMC-21-0043). Due to the retrospective nature of the study, informed consent was waived.

Preeclampsia was characterized based on the criteria established by ACOG [15]. We have outlined both the standard approach to managing preeclampsia at our medical center and the specific preeclampsia definition in accordance with the ACOG criteria in the appendix for reference (Appendix A).

This study involved a thorough review of all electronic medical files by study personnel, including obstetric, neonatal, and delivery data. Maternal, obstetric, and neonatal information was extracted from the center’s comprehensive perinatal database, which is prospectively documented upon admission and after delivery. The collected data included personal and family history, gynecologic and obstetric history, details of the current pregnancy (e.g., assisted reproductive technology, number of fetuses, gestational weight gain, and routine evaluations), pregnancy-related complications (e.g., diabetes, oligohydramnios, number and reasons for repeated hospitalizations), blood pressure values, laboratory results, and fetal status. Delivery data encompassed gestational age at delivery, mode of delivery, birth weight, and perinatal complications.

Specifically for blood pressure measurements—gestational age at first high blood pressure measurement, gestational age at first diagnosis, all follow-up blood pressure values, minimal and maximal indices of systolic and diastolic blood pressures, indices of mean arterial pressure {MAP = [systolic blood pressure + (2 × diastolic blood pressure)]/3} and pulse pressure were collected.

Parturients were then categorized into 2 groups: control or study groups based on progression to sPE. All variables were evaluated and compared between study and control groups to assess predictors for progression to sPE in gravidas presenting with GH, PE, or superimposed PE.

Data analysis was performed using SPSS version 28.0 (IBM Corp., Armonk, NY, USA). *p* < 0.05 was considered statistically significant. Univariate analysis was performed to identify statistically significant individual factors that were associated with the subsequent progression to sPE. Categorical data were analyzed using Fisher’s exact test or χ^2^ test and continuous variables were compared using the Mann–Whitney–Wilcoxon test as appropriate. Relevant factors that were found statistically significant on the univariate analysis and other previously determined known risk factors were subsequently evaluated using logistic regression analysis to control for confounders. The prediction performance of the most important factors for the progression of PE was then evaluated using ROC statistics.

## 3. Results

**Figure 1:**
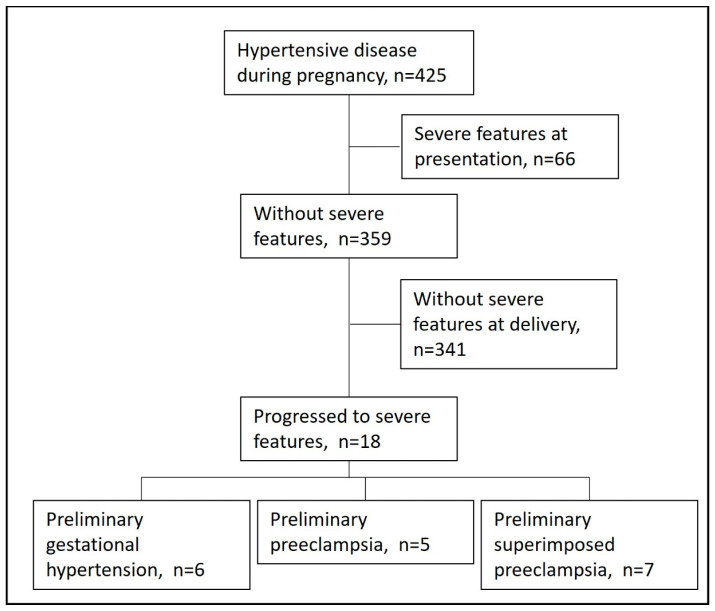
Study cohort.

Overall, during the study period, there were 13,714 deliveries. Of them, 425 (3%) had a diagnosis of any hypertensive disorder during pregnancy; 66 presented initially with sPE and thus were excluded from the analysis. Among the remained 359 women, 18 (5%) progressed to sPE (Figure 1).

Maternal and obstetrical characteristics stratified to progression to severe features are presented in Table 1.

Overall, for the entire cohort, the median maternal age was 31 years (IQR 27–36), and the median pre-pregnancy BMI was 32.8 kg/m^2^ (IQR 28.7–37.4), with a 5% pre-pregnancy smoking history. One hundred and seventy-five parturients were nulliparas (48.7%). Moreover, 3.3% of parturients had diabetes mellitus and 4.7% of them had chronic hypertension. Lastly, at presentation, 229 (63.4%) had PE without severe features, 114 (31.6%) had GH and 16 (4.5%) were diagnosed with superimposed PE.

Gravidas which eventually progressed to sPE had higher rates of maternal diabetes and chronic hypertension. Among multiparous women, the study group had higher rates of gravidas with shorter than 2 years or longer than 5 years interpregnancy interval from their previous pregnancy, and their rates of previous hypertensive disorder and especially sPE were higher.

The most common preliminary diagnosis, at first presentation, for parturients that eventually progressed to sPE was superimposed PE on chronic hypertension. Also, gravidas that progressed to sPE presented at an earlier gestational age and had a higher number of repeated admissions. Lastly, gravidas that progressed to sPE had higher rates of aspirin treatment during the current pregnancy, delivered smaller babies, at an earlier gestational age, with higher rates of cesarean deliveries (Table 2).

**Figure 2:**
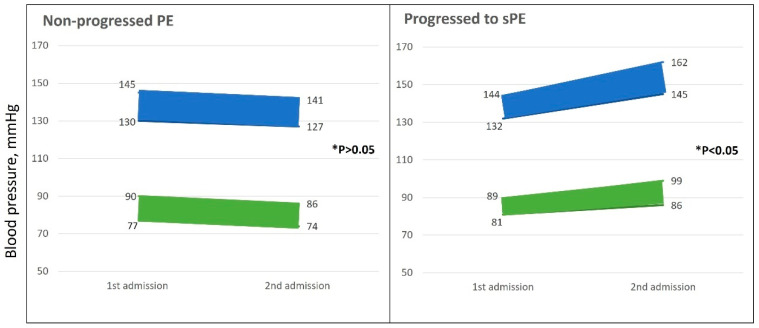
Blood pressures are presented at 1st admission and at 2nd admission (for 72 parturients). Green lines represent diastolic pressures (min and max) and blue lines represent systolic pressures (min and max). * The *p* value represents the differences in blood pressure between first and second admissions evaluated for every group separately.

A review of the blood pressure indices at first admission demonstrated no significant statistical differences between groups. Nevertheless, visualization of measurements in gravidas with recurrent admissions demonstrated a trend toward increased blood pressure indices between the first and following admission as a marker for progression to sPE (Figure 2).

Next, to adjust our results to potential confounders we used logistic regression analysis. Variables that entered the analysis included maternal diabetes, previous sPE or any other hypertension disorder during pregnancy, previous delivery within 2–5 years, gestational age at initial admission, gestational diabetes, number of admissions, and the preliminary diagnosis. Early gestational age at the initial presentation and superimposed PE as the preliminary diagnosis remained the only significant variables associated with progression to sPE (aOR 0.74 (0.56–0.97) and 34.44 (1.07–1111.85), aOR (95% CI), respectively, *p* < 0.05 for both) with combined ROC-AUC prediction performance of 0.89, 95% CI 0.83–0.95, *p* < 0.001 (Table 3).

## 4. Discussion

Hypertensive disorders during pregnancy are a common obstetric scenario, well researched, and yet still a conundrum. Even though they can be of different pathophysiology, they share the same clinical management that is targeted to reduce risks related to progression to severe features. Thus, in this study, we aimed to identify the variables that potentially predict progression to severe disease in parturients already diagnosed with mild hypertensive disorders during pregnancy.

Our main findings were as follows: 1. factors that were linked to progression to sPE included: chronic hypertension, maternal diabetes, any hypertensive disorder in a previous pregnancy, number of hospitalizations, lower gestational age at initial presentation, blood pressure trends, and superimposed PE as the preliminary diagnosis; 2. Previous delivery within 2–5 years was the only protective variable from PE progression; 3. Following regression analysis and adjustment to confounders, early gestational age at initial presentation and superimposed PE remained the only significant variables associated with PE progression to sPE.

While a wealth of data has been published regarding the prediction of PE, only a limited amount of research has endeavored to pinpoint risk factors for the progression of mild hypertensive disorders during pregnancy to sPE. Previous studies have proposed a range of risk factors for PE such as maternal age (both young and advanced), obesity, race (particularly Black or Hispanic), diabetes, chronic hypertension, renal or autoimmune diseases, nulliparity, assisted reproductive technology, and multifetal gestation [16,17,18,19,20,21]. Similar risk factors have also been suggested to be linked with sPE. However, it’s worth noting that studies evaluating these associations often focused on predicting these conditions in generally healthy populations [16,17,18,19,20,21].

With regard to women already diagnosed with hypertensive disorders during pregnancy, only several models have been developed and published with the goal of forecasting the progression to sPE. The fullPiers model was developed by von Dadelszen et al. in 2011 [12]. The model aimed to detect the variables associated with fatal or life-threatening complications in women with PE within 48 h of hospital admission. The model ultimately used 6 variables including gestational age at admission, chest pain or dyspnea, oxygen saturation (SpO_2_), platelet count, serum creatinine, and serum aspartate transaminase with ROC-AUC of 88%. The study had a strong methodology being a prospective multicenter study and demonstrated the feasibility of targeting women who eventually progressed to sPE. Nevertheless, it relied on subjective variables like chest pain or dyspnea that are not quantifiable and therefore are hard to implement in clinical practice. Also, the study included only PE as the preliminary diagnosis, and chronic hypertension, which is an established important variable for adverse outcomes was not included.

The second model was developed by Zwertbroek et al. [13]. This model was based on data from the HYPITAT -II trial [16] and included 519 women presenting with mild hypertensive disorders at 34–37 gestational weeks. In this study, 22% of women progressed to sPE [10]. Similar to our study, variables like chronic hypertension, gestational age at presentation, systolic blood pressure, creatinine levels, and lactate dehydrogenase were found discriminatory for women who progressed to sPE. However, other factors like maternal age and platelet count that entered the model in Zwertbroek’s study were not found significant in ours. The authors created a model based on all the above variables with 76% prediction performance, lower than the performance achieved in our study that was solely based on gestational age at presentation and superimposed PE. The difference in results may be attributed to the preliminary cohort evaluating only women at 34–37 gestational weeks. Moreover, the small sample size raises doubt about the generalizability of the results for both studies. Similarly, Van der Tuuk et al. assessed the HYPITAT cohort and found that with similar variables the prediction can reach 71% [14]. Other studies that evaluated risk factors for progression to sPE focused on specific subgroups: Johnston et al. focused on prediction of sPE remote from term and found that systolic blood pressure was the only variable that was associated with delivery within 7 days was systolic blood pressure [11]. Minhas et al. found that among women with chronic hypertension, elevated blood pressure and increased rate of angiogenic factors (sFlt/PlGF) were associated with progression to sPE [22].

Hypertensive disorders during pregnancy are becoming increasingly common, and their prevalence continues to rise. When these conditions progress to sPE, they pose a significant risk of perinatal morbidity and mortality [15,16]. One of the most daunting challenges for the obstetrician is identifying the pregnant individuals who will experience complications, providing them with vigilant monitoring during their hospitalization, arranging transfers to specialized care facilities, administering antenatal steroids, and considering earlier delivery. On the other hand, once we identify these women, we will be able to offer a more relaxed approach to pregnancy surveillance for the rest of the women, potentially even preventing preterm or early-term birth. As previously noted, although numerous studies have established the risk factors for PE, their overall ability to predict PE or sPE in the general population, regardless of the criteria used, has demonstrated notably low positive predictive values, ranging from 8% to 33% [23]. Therefore, the possibility of recognizing women who will progress to sPE once diagnosed with mild hypertensive disorders may potentially add important value to the current literature. Once proven feasible, future studies should refine the model, integrating angiogenic factors such as sFlt and PlGF, and validating it in a large cohort of women.

The strengths of the study lie in the meticulous review of all the electronic medical files by study personnel, and the numerous variables that were included in the initial statistical analysis including the demographic, obstetrical, and biophysical measurements at each and during every admission. Nevertheless, our study is still limited by its relatively small sample size and its retrospective nature as not all variables were available for comparison for all women in the cohort. Additionally, we recognize the vast subjectivity in the management of PE, both with and without severe features, remote from term. Moreover, due to the small sample size, we included both early- and late-onset PE in our cohort, which might have interfered with the results. Lastly, the single-site institution may limit the applicability of results to the broader population.

## 5. Conclusions

In conclusion, according to our study, early gestational age at presentation and superimposed PE as the preliminary diagnosis are the only independent factors that are associated with progression to sPE in women already diagnosed with hypertensive disorders during pregnancy. Nevertheless, our results should be taken with caution due to the study’s limitations. Further studies should assess and validate models predicting sPE thus directing more stringent follow-up and even earlier delivery to prevent adverse perinatal outcomes.

## Figures and Tables

**Table 1:** Study cohort characteristics stratified by progression to preeclampsia with severe features.

Variable	Progressed to sPE n = 18	Non-Progressed Hypertensive Disease n = 341	*p*-Value
Maternal variables:			
Maternal age, years	34 (26.8–37.5)	31 (27.0–35.0)	0.233
BMI, Kg/m^2^	34.3 (27.7–40.1)	32.8 (28.7–37.1)	0.578
Smoking	0 (0.0)	17 (5.0)	1.000
Thrombophilia	0 (0.0)	8 (2.3)	1.000
Chronic hypertension	7 (38.9)	10 (2.9)	<0.001
Diabetes mellitus	4 (22.2)	8 (2.4)	0.002
Thyroid disease	2 (11.1)	22 (6.5)	0.342
Obstetric variables:			
Nulliparity	8 (44.4)	167 (49.0)	0.811
Previous delivery interval between 2–5 years	2 (22.2)	100 (65.4)	0.013
Previous pregnancy hypertensive disorder	7 (70.0)	52 (29.5)	0.012
Previous sPE	4 (40.0)	22 (12.6)	0.037
Previous nsPE	3 (30.0)	30 (17.0)	0.386
Pregnancy variables:			
Fertility treatment	4 (22.2)	37 (10.9)	0.138
Smoking in pregnancy	0 (0.0)	15 (4.4)	1.000
Twin gestation	2 (11.1)	12 (3.5)	0.151
Aspirin treatment	7 (38.9)	40 (11.7)	0.004
PAPP-A ≤ 0.4, MoM	2 (28.6)	28 (13.9)	0.264
HCG ≥ 3, MoM	1 (12.5)	12 (5.6)	0.386
AFP ≥ 3, MoM	0 (0.0)	1 (0.5)	1.000
Gestational diabetes	5 (27.8)	29 (8.5)	0.020
Oligohydramnios	0 (0.0)	8 (2.3)	1.000
Preliminary diagnosis at 1st presentation:			
Gestational hypertension	6 (33.3)	108 (31.7)	<0.001
Preeclampsia	5 (27.8)	224 (65.7)	
Superimposed preeclampsia	7 (38.9)	9 (2.6)	
1st admission variables:			
GA at 1st presentation, weeks	32.5 (26.3–35.3)	38.0 (36.0–39.0)	<0.001
Proteinuria (≥300mg/24-h) at 1st presentation (%)	4 (26.7)	55 (33.5)	0.776
1st admission blood pressure:			
Min diastolic, mmHg	79.0 (74.0–88.5)	79.0 (68.0–86.0)	0.275
Max diastolic, mmHg	91.5 (79.8–99.0)	91.5 (83.0–96.0)	0.947
Min systolic, mmHg	131.5 (122.5–144.0)	131.0 (123.0–139.0)	0.752
Max systolic, mmHg	141.0 (133.0–155.0)	145.0 (139.0–152.0)	0.492
MAP, mmHg	121.0 (116.8–132.1)	124.0 (117.0–130.0)	0.590
PP, mmHg	56.5 (49.5–64.3)	58.0 (50.0–65.0)	0.620
1st admission Labs:			
Creatinine level	0.50 (0.40–0.60)	0.60 (0.50–0.60)	0.040
Platelet level	210.5 (143.5–273.0)	206.5 (173.8–250.0)	0.984
Elevated liver enzymes	1 (5.6)	17 (5.2)	1.000
Uric acid	3.7 (3.3–4.8)	4.5 (3.8–5.3)	0.030
Alkaline phosphatase	113.5 (84.0–140.8)	144.5 (116.0–180.3)	<0.001
Number of admissions	2 (1–2)	0 (0–1)	<0.001

Categorical data are presented as numbers (%) continuous variables are presented as medians (interquartile range). Significant differences are presented in bold (if *p*-value < 0.05). sPE—severe preeclampsia; nsPE—non-severe preeclampsia; BMI—body mass index; PAPP-A—pregnancy-associated plasma protein A; MoM—multiple of the median; HCG—human chorionic gonadotropin; AFP—alpha-fetoprotein; GA—gestational age; MAP—mean arterial pressure; PP—pulse pressure.

**Table 2:** Birth outcome stratified by progression to preeclampsia with severe features.

Variable	Progressed to sPE n = 18	Non-Progressed Hypertensive Disease n = 341	*p*-Value
Birth outcome:			
GA at delivery, weeks	36.0 (33.0–37.0)	38.0 (37.0–39.0)	<0.001
GA at delivery, weeks			<0.001
GA ≤ 28	0 (0)	0 (0)	
GA 28–32	3 (16.7)	4 (1.2)	
GA 32–37	12 (66.7)	111 (32.6)	
GA > 37	3 (16.7)	225 (66.2)	
Birth weight, grams *	2656.5 (2111.3–3772.5)	3169.0 (2836.3–3527.5)	0.129
Cesarean delivery	10.0 (55.6)	89.0 (26.1)	0.012
Neonate admission length, days	6.0 (5.3–17.8)	4.0 (3.0–5.0)	<0.001

Categorical data are presented as numbers (%), continuous variables are presented as medians (interquartile range). Significant differences are presented in bold (if *p*-value < 0.05). * Birthweight is calculated for singleton pregnancies. sPE—severe preeclampsia; GA—gestational age.

**Table 3:** Logistic regression analysis demonstrating predictors for progression to preeclampsia with severe features.

Variable	Crude OR	Adjusted OR	95% Confidence Interval	*p*-Value
Diabetes mellitus	11.82	0.21	0–14.68	0.475
Previous severe preeclampsia	4.61	1.21	0.09–16.33	0.887
Previous pregnancy hypertensive disorder	5.56	1.85	0.10–34.11	0.680
Previous delivery interval between 2–5 years	0.15	0.08	0–1.03	0.053
Gestational diabetes	4.14	3.86	0.23–63.69	0.377
GA at 1st presentation, weeks	0.75	0.74	0.56–0.97	0.027
Number of admissions	2.80	1.15	0.44–3.01	0.778
Preliminary diagnosis at 1st presentation				
Gestational hypertension	Referent	-	-	-
Non-severe preeclampsia	0.40	3.78	0.23–63.69	0.356
Superimposed preeclampsia	14.00	34.44	1.07–1111.85	0.046

GA—gestational age; OR—odds ratio.

## Data Availability

The data presented in this study are available on request from the corresponding author. The data are not publicly available due to privacy restrictions.

## Appendix A

### Appendix A.1. Preeclampsia Criteria

Hypertensive disorders during pregnancy were defined according to the American College of Obstetrics and Gynecology (ACOG) criteria [12]. Gestational hypertension was defined as a systolic blood pressure of 140 mm Hg or higher or a diastolic blood pressure of 90 mm Hg or higher, or both, on two occasions at least 4 h apart after 20 weeks of gestation in a woman with a previously normal blood pressure without proteinuria. Preeclampsia was defined as any gestational hypertension with accompanying proteinuria (300 mg or more per 24-h urine collection or urine protein/creatinine ratio of 0.3 mg/dL or more or dipstick reading of 2+) or if severe features are present, even in the absence of proteinuria. Superimposed Preeclampsia on chronic hypertension was defined as worsening blood pressure or new onset proteinuria after 20 weeks of gestation.

Preeclampsia with severe features was defined as any of the following: systolic blood pressure of 160 mm Hg or more, or diastolic blood pressure of 110 mm Hg or more on two occasions at least 4 h apart (unless antihypertensive therapy was initiated before this time); new-onset headache unresponsive to medication and not accounted for by alternative diagnoses; visual disturbances; severe persistent right upper quadrant or epigastric pain unresponsive to medications; thrombocytopenia (platelet count less than 100 × 10^9^/L); an impaired liver function that is not accounted for by alternative diagnoses and as indicated by abnormally elevated blood concentrations of liver enzymes (to more than twice the upper limit normal concentrations); renal insufficiency (serum creatinine concentration more than 1.1 mg/dL or a doubling of the serum creatinine concentration in the absence of other renal diseases); pulmonary edema; and/or eclampsia.

### Appendix A.2. The Standard Approach to Preeclampsia

Women presenting for the first time at the obstetric emergency room with suspected preeclampsia are admitted for evaluation. Patient’s symptoms and vital signs are monitored at least 3 times a day, labs (including complete blood count, creatinine level, uric acid, electrolytes, liver enzymes, LDH level, and coagulation function), and a 24-h urine collection is performed at least once during hospitalization. As for fetal status, a fetal monitor is maintained at least twice a day, and a sonographic estimated fetal weight is performed. If severe features appear and a woman is defined as sPE, a decision to deliver or expectedly manage the pregnancy is taken according to gestational age and accepted guidelines. After the complete evaluation, women are discharged to ambulatory care or hospitalized by the attending physician’s decision. Women with any diagnosis of preeclampsia, gestational hypertension, or superimposed preeclampsia without severe features are finally delivered at 37 gestational weeks provided there is no other indication for an earlier delivery.
